# O-RADS US Version 2022 Improves Patient Risk Stratification When Compared with O-RADS US Version 2019

**DOI:** 10.1148/radiol.242200

**Published:** 2025-03-18

**Authors:** Roni Yoeli-Bik, Jacques S. Abramowicz, Kristen Wroblewski, Leonhard Donle, Ryan E. Longman, Ernst Lengyel

**Affiliations:** ^1^Department of Obstetrics and Gynecology, The University of Chicago, 5841 S Maryland Ave, MC 2050, Chicago, IL 60637; ^2^Department of Public Health Sciences, The University of Chicago, Chicago, Ill

**Figure:**
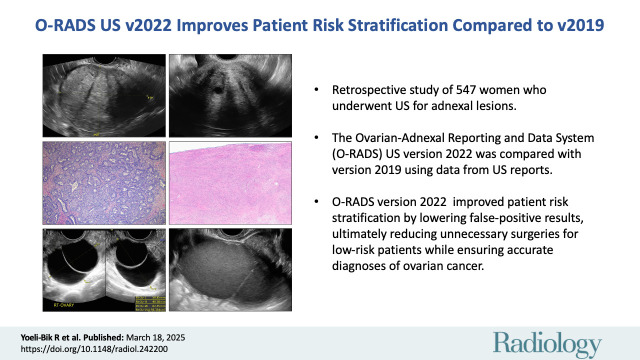

**Summary**

The Ovarian-Adnexal Reporting and Data System US version 2022 improves patient risk stratification by lowering false-positive results, ultimately reducing unnecessary surgeries for low-risk patients while ensuring accurate diagnoses of ovarian cancer.

## Introduction

The Ovarian-Adnexal Reporting and Data System (O-RADS) for US was first introduced in 2019 to provide reproducible standards for stratifying adnexal lesions into malignancy risk categories, supporting consistent documentation and evidence-based management guidelines (1). Because of the high lethality of ovarian cancer, O-RADS was designed to reduce false-negative reads by optimizing sensitivity for detecting malignancy at the expense of specificity (1). It was adopted widely in the United States, distinguishing benign from malignant adnexal lesions with a pooled sensitivity of 95.6% and specificity of 76.6% (2). However, the observed overestimation of malignancy offered an opportunity for improved scoring (3,4), leading to the O-RADS version 2022 guidelines (5). The updated system includes additional sonographic features such as bilocular lesions and acoustic shadowing for smooth solid lesions, expanded lexicon definitions for classic benign lesions, and updated management guidelines in line with established clinical recommendations. Because external validations of O-RADS version 2022 and comparison with version 2019 are still limited, this study aimed to compare their diagnostic performance with use of an independent dataset.

## Materials and Methods

This retrospective single-center diagnostic accuracy study approved by the institutional review board and compliant with the Health Insurance Portability and Accountability Act was performed at the University of Chicago Medical Center. It was based on a largely consecutive patient cohort with adnexal lesions and available US examinations (2017–2022) (6), which has since been updated with 15 more months of patient registration (November 2022–January 2024). Inclusions were patients managed surgically (within 180 days from their sonogram) or conservatively. Exclusion was follow-up less than 1 year. Adequate follow-up was defined as follows: *(a)* the mass resolved, *(b)* size decreased by at least 10%, *(c)* the mass remained unchanged over 1 year, or *(d)* the mass was identified as a classic lesion at MRI or CT (6).

Most sonographic evaluations were conducted at the study institution using GE HealthCare Voluson E8 and E10 and Samsung Elite WS80 US machines, and additional US imaging was performed at affiliated facilities. US was performed by experienced sonographers and systematically reviewed by a US researcher (R.Y.B.) with a consensus of expert US examiners (J.S.A. and R.E.L., with >40 and >20 years of experience, respectively) on about 30% of cases, providing an audit for the accuracy and quality imaging assessment using all available images, including cine clips. If a patient had multiple masses, then the one with the most suspicious characteristics was recorded for the study. Risk scores determined with O-RADS US version 2022 were assessed from previously collected sonographic variables. The observed malignancy prevalence at each risk score category and the area under the receiver operating characteristic curve were calculated for each version. Sensitivity, specificity, positive predictive value, negative predictive value, and accuracy were calculated at the 10% cutoff (O-RADS US scores 2–3 vs 4–5) using Stata 18 (StataCorp). A priori calculations indicated an 85% power to detect specificity of 69% for version 2019 versus 75% for version 2022 with 547 patients, assuming 19% malignancy prevalence, α of .05, and correlation of 0.56.

## Results

In total, 547 patients were included, with a mean age of 46 years ± 15 (SD); 43% (236 of 547) were postmenopausal, 83% (455 of 547) were managed surgically, and 17% (92 of 547) were managed conservatively. There was a malignancy prevalence of 19% (102 of 547). The most common premenopausal benign lesions were endometriomas (34% [72 of 210]), and the most common malignancies were serous borderline tumors (27% [eight of 30]). The most common postmenopausal benign lesions were serous cystadenomas (17% [24 of 143]) and cystadenofibromas (17% [24 of 143]), and the most common malignancies were high-grade serous carcinomas (31% [22 of 72]). With use of O-RADS US versions 2019 and 2022 to stratify patients into risk categories, the observed malignancy prevalence distribution remained consistent with the targeted risk (Table 1). At the same time, specificity and accuracy significantly increased with O-RADS version 2022 (Table 2). The area under the receiver operating characteristic curve for O-RADS US versions 2019 and 2022 was 0.901 (95% CI: 0.875, 0.927) and 0.905 (95% CI: 0.879, 0.930), respectively. However, with a lesion-by-lesion analysis, 29 of 547 patients (5.3%) were reclassified as lower-risk with O-RADS US version 2022 compared with version 2019. Reclassification was correct in 28 of 29 patients (97%); 16 patients moved from risk score 3 to 2 and 13 from 4 to 3. One 80-year-old patient with a malignant lesion was incorrectly shifted from risk score 4 to 3 based on O-RADS US version 2022. She had a 10.5-cm left solid adnexal lesion with regular external borders, acoustic shadowing, and minimal flow at color Doppler imaging and a CA125 level of 246 U/mL (Figure). Histopathologic examination revealed an International Federation of Gynecology and Obstetrics (ie, FIGO) stage IA low-grade ovarian endometrioid adenocarcinoma arising from an endometrioid adenofibroma. Evaluation of the patient integrating clinical and laboratory findings indicated surgery regardless of the O-RADS score. Risk models, while helpful, cannot replace clinical judgment.

**Table 1:**
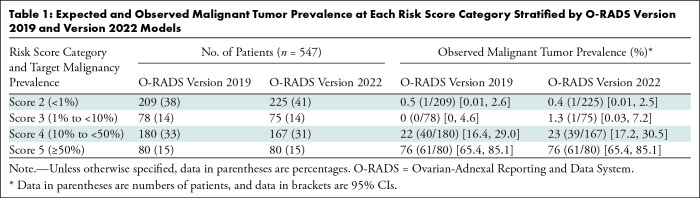
Expected and Observed Malignant Tumor Prevalence at Each Risk Score Category Stratified by O-RADS Version 2019 and Version 2022 Models

**Table 2:**
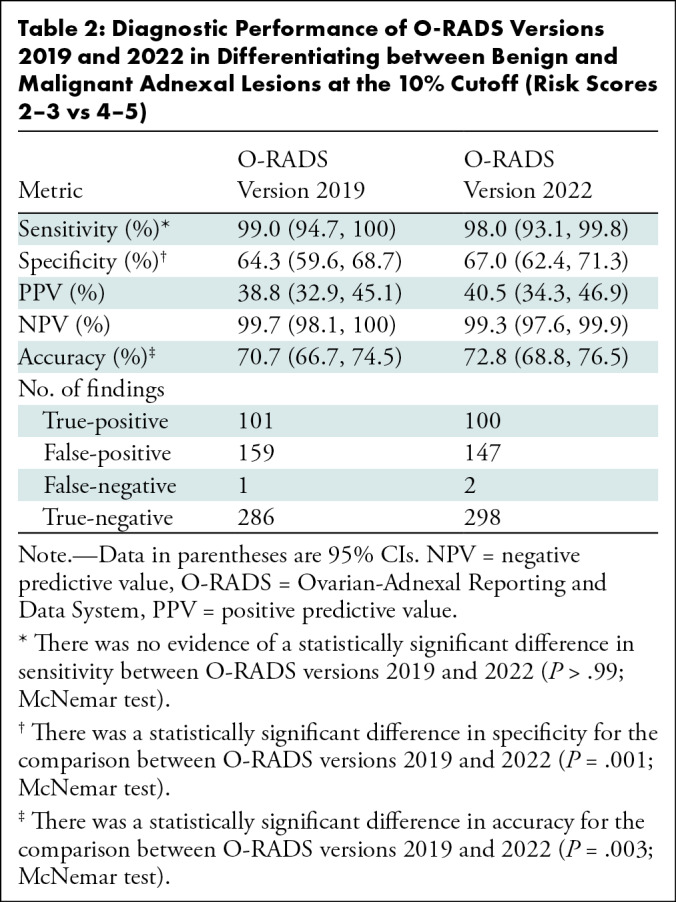
Diagnostic Performance of O-RADS Versions 2019 and 2022 in Differentiating between Benign and Malignant Adnexal Lesions at the 10% Cutoff (Risk Scores 2–3 vs 4–5)

**Figure 1:**
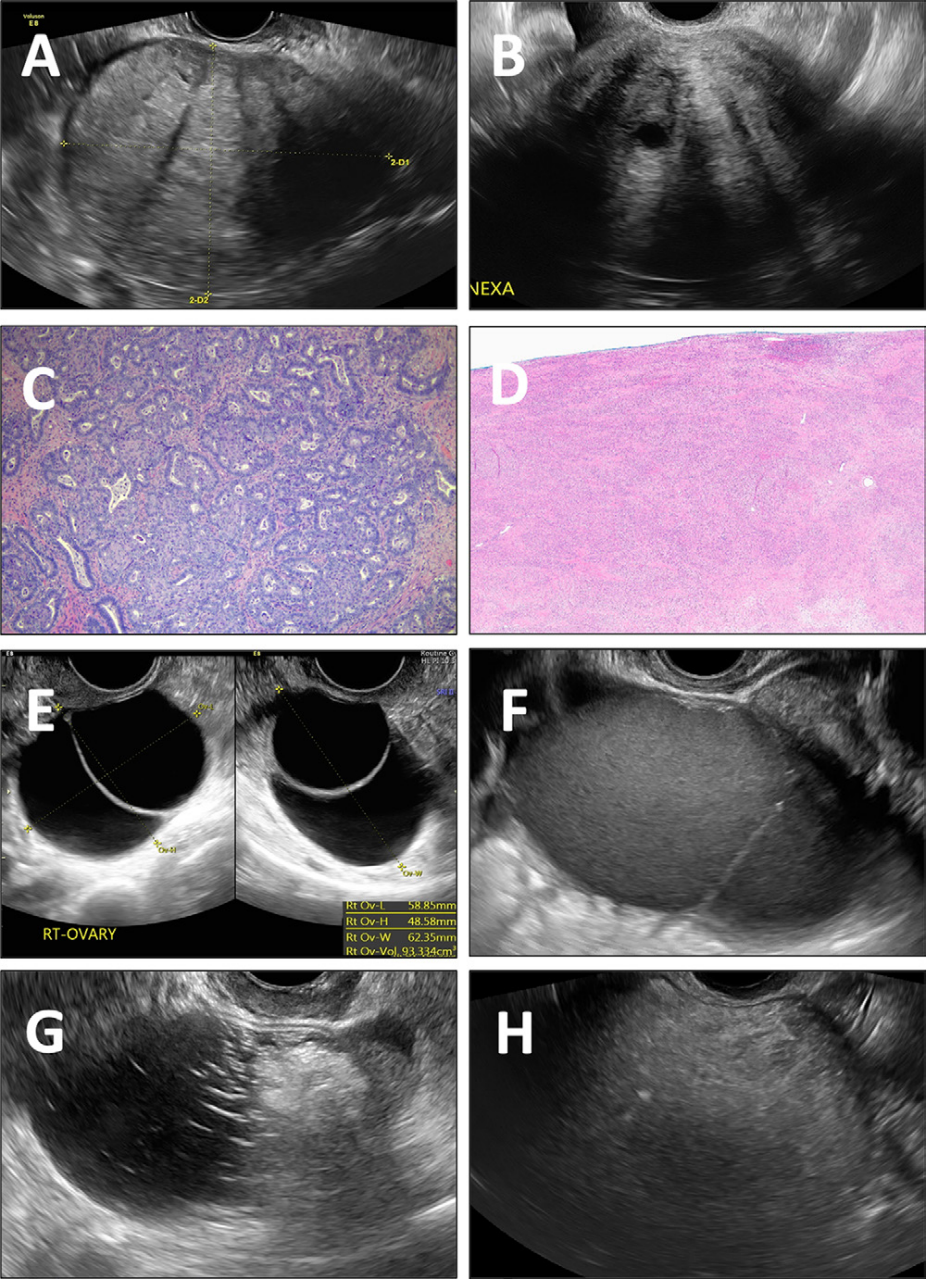
Sonographic features characterized in Ovarian-Adnexal Reporting and Data System (O-RADS) version 2022 as low risk of malignancy. **(A)** US image in an 80-year-old patient shows a smooth solid adnexal mass with acoustic shadowing and a minimal color score of 2 (not shown). Pathologic findings showed a low-grade International Federation of Gynecology and Obstetrics (ie, FIGO) stage IA ovarian endometrioid adenocarcinoma (10.5 cm) arising in the background of endometrioid adenofibroma. This was the only case incorrectly classified as lower risk using O-RADS version 2022 compared with O-RADS version 2019. **(B)** US image in a 52-year-old patient shows a right smooth solid adnexal mass with acoustic shadowing and a minimal color score (not shown). Histologic findings indicated an ovarian fibroma (10 cm). **(C)** Hematoxylin and eosin–stained slide of an ovarian endometrioid carcinoma shows crowded glandular structures with extensive morular differentiation embedded in dense fibrotic stroma (magnification, 100×). **(D)** Hematoxylin and eosin–stained slide of an ovarian fibroma consists of monotonous sheets of spindled stromal cells (magnification, 40×). **(E)** US images in a 61-year-old patient shows a right bilocular cystic lesion with smooth walls, which turned out to be an ovarian rete cystadenoma (5 cm). **(F)** US image in a 24-year-old patient shows a left bilocular cystic lesion with ground-glass echogenicity and small peripheral echogenic foci (endometrioma). **(G)** US image in a 47-year-old patient shows a right unilocular cystic lesion with a hyperechoic component with regional shadowing and hyperechoic lines and dots (mature cystic teratoma). **(H)** US image in a 63-year-old patient shows a right unilocular hyperechoic cystic lesion with diffuse shadowing (mature cystic teratoma). Of note, the figure does not review all new changes in O-RADS version 2022 (eg, additional characteristics of classic benign lesions).

## Discussion

This study showed that both O-RADS versions were effective for stratifying patients into malignancy risk scores with high sensitivity and negative predictive value at the 10% risk threshold. The observed malignancy prevalence matched the expected targeted ranges of the O-RADS system. The malignancy proportion for O-RADS score 2 was below 1%, but the upper bound of the CIs for this risk category exceeded 1%, and the malignancy rate in O-RADS score 3 was at the lower end of the targeted range (1%–10%), as previously reported (7). O-RADS version 2022 provided better patient risk score allocation because fewer patients received false-positive results. The improved diagnostic accuracy of O-RADS version 2022 is likely driven by the addition of features that suggest benign masses: bilocular cystic lesions, acoustic shadowing with smooth solid lesions and moderate to no color flow, and more detailed descriptors of classic benign lesions. Cystic lesions with a single smooth septation have been correlated with benign origin and carry a lower risk of malignancy (5). Adnexal lesions misclassified as malignant are often fibromatous, which can manifest as solid hypoechoic tumors with acoustic shadowing—a helpful sonographic feature to correctly classify them (5,7).

Limitations of our study include a single-center retrospective study design performed at an academic center with high malignancy prevalence (19% [102 of 547 patients]) and the retrospective assessment of O-RADS version 2022 scores from previously collected variables. Last, only one reader initially read the US images, but 30% of all cases were reviewed by a second reader, and all US reviewers discussed indeterminate cases to reach a consensus.

In conclusion, O-RADS US version 2022 is superior to version 2019. It maximizes sensitivity while significantly improving the specificity of lower-risk lesions based on the inclusion of acoustic shadowing, bilocular cystic lesions, and more specific lexicon definitions. It may also provide improved patient management recommendations in accordance with current clinical guidelines.
